# Benzidin und seine Salze: MAK-Begründung – Erratum

**DOI:** 10.34865/mb9287d11_2err1

**Published:** 2024-06-28

**Authors:** Andrea Hartwig, MAK Commission

**Affiliations:** 1 Institut für Angewandte Biowissenschaften, Abteilung Lebensmittelchemie und Toxikologie, Karlsruher Institut für Technologie (KIT) Adenauerring 20a, Geb. 50.41 76131 Karlsruhe Deutschland; 2 Ständige Senatskommission zur Prüfung gesundheitsschädlicher Arbeitsstoffe, Deutsche Forschungsgemeinschaft, Kennedyallee 40, 53175 Bonn, Deutschland. Weitere Informationen: Ständige Senatskommission zur Prüfung gesundheitsschädlicher Arbeitsstoffe | DFG

**Keywords:** Benzidin und seine Salze, Humankanzerogen, Harnblasenkrebs, Kanzerogenität, Keimzellmutagenität, Hautresorption, Wirkungsmechanismus

## Abstract

The production and technical use of benzidine [92-87-5] and its salts are prohibited by law in the EU and many other countries. Although benzidine was added to the List of MAK and BAT Values in 1966 and classified in Carcinogen Category 1 in 1975, documentation for the substance had not yet been compiled. To remedy this, the German Senate Commission for the Investigation of Health Hazards of Chemical Compounds in the Work Area (MAK Commission) has now prepared the documentation and additionally evaluated the end points germ cell mutagenicity, sensitization and skin absorption. Relevant studies and reviews were identified from a literature search. The carcinogenicity of benzidine has not been re-evaluated in detail because there is unequivocal evidence of its carcinogenic potential in humans: Numerous case reports and epidemiological studies from different countries show a strong and consistent association between benzidine exposure and the risk of bladder cancer. Many studies found benzidine to be genotoxic in vitro and in vivo. No studies with germ cells are available. Findings that benzidine dihydrochloride crosses the blood-brain barrier in mice after oral administration suggest that the substance may also be able to pass through the blood-testis barrier. Therefore, benzidine and its salts have been classified in Category 3 A for germ cell mutagens. Benzidine is readily absorbed by rats after dermal application in acetone. The substance is a genotoxic carcinogen and a systemically tolerable dose cannot be derived. Therefore, the designation with "H" (for substances which can be absorbed in toxicologically relevant amounts) has been retained. No studies that investigated benzidine salts are available. Although the salts are probably absorbed less readily, absorption cannot be ruled out and the salts of benzidine also remain designated with "H". There are no studies that investigated the sensitizing effects of benzidine in animals. Studies using alternative test methods are also not available. A limited number of clinical findings show positive reactions to benzidine. As patients with sensitization to aromatic para-amino compounds, such as p-phenylenediamine, often react to other compounds of this substance class as well, cross-reactivity to benzidine is possible. The data available are too limited to draw a conclusion regarding the skin sensitizing potential of benzidine and its salts. Data for respiratory sensitization are not available.

Erratum für:
Hartwig A, MAK Commission. Benzidin und seine Salze. MAK-Begründung. MAK Collect Occup Health
Saf. 2024 Jun;9(2):Doc028. 10.34865/mb9287d9_2or

## Anmerkung

In der Originalversion des Artikels (DOI 10.34865/mb9287d9_2or) sind in Kapitel 3 „Toxikokinetik und Metabolismus“ falsche Zahlenwerte genannt, die in diesem Erratum korrigiert wurden.

**Table:** 

**MAK-Wert**	**–**
**Spitzenbegrenzung**	**–**
	
**Hautresorption (1966)**	**H**
**Sensibilisierende Wirkung**	**–**
**Krebserzeugende Wirkung (1975)**	**Kategorie 1**
**Fruchtschädigende Wirkung**	**–**
**Keimzellmutagene Wirkung (2023)**	**Kategorie 3 A**
	
**EKA**	**nicht festgelegt**
	
Synonyma	4,4′-Bianilin (1,1′-Biphenyl)-4,4′-diamin 4,4′-Diaminodiphenyl 4,4′-Diphenylendiamin
Chemische Bezeichnung (IUPAC-Name)	4-(4-Aminophenyl)anilin
CAS-Nr.	92-87-5
Formel	Strukturformel von Benzidin. 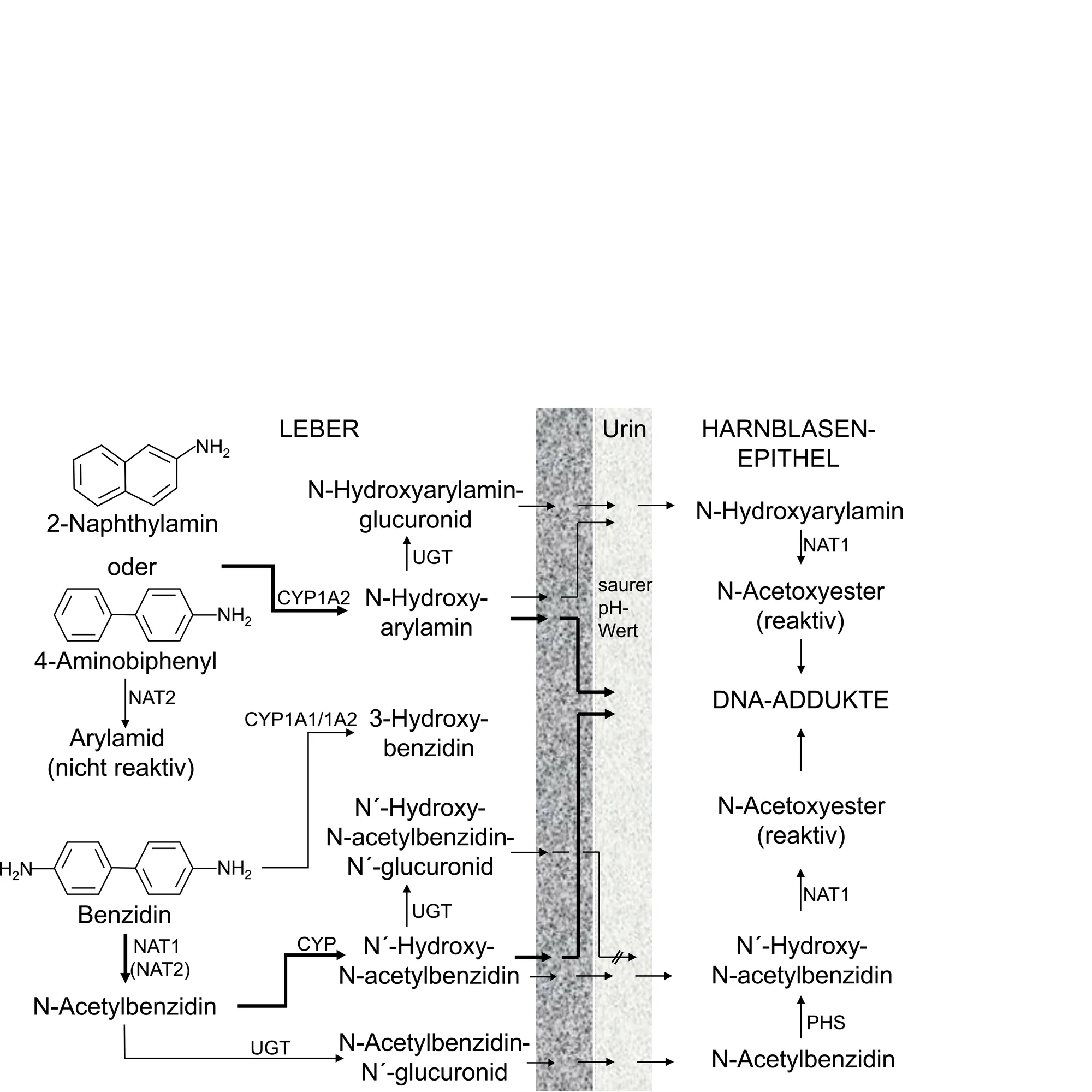
	C_12_H_12_N_2_
Molmasse	184,24 g/mol
Schmelzpunkt	127–128 °C (Lewalter 2000); 120 °C (NTP 2021)
Siedepunkt bei 987 hPa	401 °C (Lewalter 2000; NTP 2021)
Dichte bei 20 °C	1,25 g/cm^3^ (Lewalter 2000; NTP 2021)
Dampfdruck	6,67 × 10^–4^ hPa bei 20 °C (Lewalter 2000); 11,97 × 10^–7^ hPa bei 25 °C (NTP 2021)
log K_OW_	1,34 (Lewalter 2000; NTP 2021)
Löslichkeit	322 mg/l Wasser (NTP 2021)
	
Hydrolysestabilität	k. A.
Einsatzverbote und Verwendung	Die Herstellung und technische Verwendung von Benzidin oder seinen Salzen ist durch Rechtsvorschriften der EU sowie auch in mehreren anderen Ländern (z. B. Japan, Kanada und Schweiz) verboten. In den USA wird Benzidin seit dem Jahr 1976 nicht mehr in großem Umfang für kommerzielle Zwecke hergestellt, obwohl kleine Mengen für diagnostische Tests weiterhin verfügbar sind. Es wird in Deutschland, der Sonderverwaltungsregion Hongkong, Indien, der Volksrepublik China, der Schweiz und den USA in geringen Mengen für die Forschung hergestellt und/oder geliefert. Über die Herstellung und Verwendung von Benzidin in der Farbstoffproduktion wird aus einigen Entwicklungsländern berichtet, ebenso wie über die Verlagerung der Benzidin-Produktion aus anderen europäischen Ländern nach Serbien und Montenegro (IARC 2012).

Seit dem Jahr 1994 ist in Deutschland die Verwendung bestimmter Azofarbstoffe in Konsumgütern, die in direktem und längerem Kontakt mit der menschlichen Haut stehen (z. B. Kleidung, Bettwäsche, Schuhe, Handschuhe usw.) verboten. Betroffen sind Farbstoffe, die nach der Reduktion einer oder mehrerer Azogruppen eines oder mehrere von 22 spezifischen aromatischen Aminen (einschließlich Benzidin) in nachweisbaren Konzentrationen (d. h. > 30 mg/kg) freisetzen können. Im Jahr 2002 wurde dieses Verbot EU-weit ausgesprochen und betraf auch Produkte, die mit der Mundhöhle in Berührung kommen. Die FDA (US Food and Drug Administration) hat den Benzidingehalt in Lebensmittelfarbstoffen auf 1 µg/kg begrenzt (IARC 2012). Das zuvor erwähnte EU-weite Verbot ist seit Mitte 2009 in Anhang XVII (Ziffer 43) der REACH-Verordnung geregelt. Tätowierfarben dürfen gemäß der REACH-Verordnung maximal 5 µg Benzidin/kg enthalten (Europäisches Parlament und Europäischer Rat 2006).

Es liegen bisher eine BAT-Begründung (Lewalter 2000) und ein BAT-Addendum (Nasterlack 2010) vor. 

Obgleich Benzidin seit 1966 in der MAK- und BAT-Werte-Liste geführt wird und seit 1975 in Kanzerogenitäts-Kategorie 1 eingestuft ist, gab es bislang keinen Begründungstext. Um diese Publikationslücke zu schließen, wurde die vorliegende Begründung verfasst. Eine Neubewertung der Kanzerogenität von Benzidin erfolgt dabei nicht, da die humankanzerogene Wirkung in vielen Untersuchungen belegt ist. Die Endpunkte Keimzellmutagenität, Hautresorption und sensibilisierende Wirkung werden in dieser Begründung erstmals bewertet.

Die Begründung basiert vorwiegend auf Reviews (ATSDR 2001, 2009; Carreón et al. 2006; IARC 2012; Lewalter 2000; Nasterlack 2010; NTP 1980, 2021) und Zusammenfassungen zu den bewertungsrelevanten Endpunkten. 

Das aromatische Amin Benzidin gilt wie 2-Naphthylamin und 4-Aminobiphenyl als nachweislich für den Menschen kanzerogen. Die kanzerogene Wirkung wurde in Form von „Harnblasenkrebs" zwar schon um das Jahr 1900 bei Anilin-Arbeitern erkannt, aber jahrzehntelang falsch gedeutet. So konnten die kontroversen Diskussionen um die Ursache von erhöhten Harnblasentumor-Inzidenzen bei gegen Aminoaromaten exponierten Beschäftigten lange nicht geklärt werden. Selbst als im Jahr 1927 die Harnblasentumore bei Beschäftigten einer Farbstofffabrik eindeutig auf den Benzidin-Umgang zurückgeführt wurden, wurden diese Beobachtungen noch als Konsequenzen typischer Mischexpositionen interpretiert. Diese Bewertung wurde auch durch die Ergebnisse zahlreicher Folgestudien nicht entscheidend beeinflusst, zumal der tierexperimentelle Nachweis der kanzerogenen Wirkung des Benzidins an Maus und Ratte nicht gelang. Selbst die im Jahr 1950 gezeigten Harnblasentumore an Benzidin-belasteten Hunden führten lediglich zu einer Verbesserung der Hygienebedingungen in der Produktion, aber zu keinem generellen Herstellungs- und Umgangsverbot für Benzidin. Die verbesserten Hygienebedingungen konnten aufgrund der jahrzehntelangen Latenzzeit von der Exposition bis zur Manifestation der Wirkung die Harnblasentumor-Erkrankungsraten kurzfristig nicht beeinflussen. Erst im Jahr 1962 wurde in England, im Jahr 1967 dann in Deutschland und anderen westlichen Ländern die Benzidin-Produktion eingestellt. Weltweit, aber vorzugsweise in Fernost, wurden im Jahr 1972 noch 10 000 und im Jahr 1983 sogar noch 15 000 Jahrestonnen aller Diphenylbasen, u. a. auch Benzidin, hergestellt. Der Umgang mit Benzidin wurde erst nach 1978 drastisch eingeschränkt, als die Bioverfügbarkeit von Benzidin in Stoffwechseluntersuchungen verschiedener Benzidinfarbstoffe zunächst tierexperimentell und später auch im Harn von Arbeitern nachgewiesen werden konnte (Lewalter 2000).

## 1 Allgemeiner Wirkungscharakter

Benzidin wird inhalativ und besonders auch dermal schnell resorbiert und in Form des freien oder konjugierten Amins und konjugierter Metaboliten mit einer Halbwertszeit von bis zu sieben Stunden über die Nieren ausgeschieden.

Die krebserzeugende Wirkung von Benzidin beim Menschen ist erwiesen. Die Tumore der ableitenden Harnwege werden im Allgemeinen nach langjährigem Benzidin-Umgang mit Latenzzeiten von 20 Jahren beobachtet.

Zahlreiche Studien belegen die genotoxische Wirkung von Benzidin in vitro und in vivo in Form von Mutationen, Klastogenität und Aneugenität. Es liegen keine Studien an Keimzellen vor. Die Überwindung der Blut-Hirn-Schranke bei Mäusen nach oraler Gabe von Benzidindihydrochlorid spricht auch für eine Passage der Blut-Testes-Schranke. Somit ist davon auszugehen, dass Benzidin oder seine Metaboliten die Keimzellen erreichen. 

Zur sensibilisierenden Wirkung von Benzidin liegen nur wenige Studien vor. Die klinischen Befunde zeigen, dass positive Reaktionen auf Benzidin auftreten können. Da Patienten mit einer bestehenden Sensibilisierung gegen aromatische para-Aminoverbindungen, wie p-Phenylendiamin, häufig auch auf andere Verbindungen dieser Substanzklasse reagieren, ist eine Kreuzreaktivität auf Benzidin möglich.

## 2 Wirkungsmechanismus

### Entstehung der Harnblasentumore

Benzidin wird in Form des freien oder konjugierten Amins und konjugierter Metaboliten über die Nieren ausgeschieden. Im sauren Urin entstehen stark elektrophile Intermediate, was zur Bildung von DNA-Addukten in den Zellen des Urothels führt und als die tumorinitiierende Wirkung des Benzidins in der Harnblase angesehen wird (Carreón et al. 2006, 2007).

#### Entgiftung und Einfluss des Acetyliererstatus

Studien zum Benzidin-Metabolismus zeigen, dass Benzidin, welches zu den Diarylaminen gehört, anders metabolisiert wird als die aromatischen Monoarylamine (wie 2-Napthylamin und 4-Aminobiphenyl). Insbesondere scheint die Acetylierung durch die N-Acetyltransferase (NAT1 und NAT2), im Gegensatz zum Metabolismus von Monoarylaminen, für Benzidin kein Entgiftungsschritt zu sein. *NAT2* ist polymorph, das heißt, ein Fehlen von zwei vollständig funktionalen Allelen führt bei den betroffenen Personen zu einer verminderten NAT-Enzymaktivität, sie sind langsame Acetylierer. Bei Exposition gegen Monoarylamine bilden langsame Acetylierer höhere Mengen an N-Hydroxyarylaminen als schnelle Acetylierer. N-Hydroxyarylamine werden als die Initiatoren der kanzerogenen Wirkung angesehen. Langsame Acetylierer hingegen haben bei einer Exposition gegen Diarylamine wie Benzidin kein erhöhtes Krebsrisiko in der Harnblase (Carreón et al. 2006): Bei Benzidin erfolgt die Bildung von reaktiven Metaboliten mit elektrophilen Eigenschaften, welche für die Bildung von DNA- und Hämoglobin-Addukten und die daraus resultierenden Harnblasentumore verantwortlich gemacht werden, vor allem durch diejenigen Benzidin-Metaboliten, die zunächst durch N-Acetylierung von Benzidin an der einen Aminogruppe via NAT und anschließender oxidativer Verstoffwechselung an der zweiten Aminogruppe entstehen (Carreón et al. 2006; Pesch et al. 2013). Die ausschließliche oxidative Verstoffwechselung beider Aminogruppen durch Cytochrom-P450-Enzyme (CYP) führt zur Entgiftung (siehe Abbildung 1). Somit ist die N-Acetylierung für Benzidin kein Entgiftungsmechanismus, sondern vermutlich ein wichtiger Aktivierungsschritt. Sowohl Benzidin als auch N-Acetylbenzidin werden nach hepatischer Glucuronidierung oder Hydroxylierung in das Harnblasenlumen ausgeschieden und im Urin aufgrund des sauren pH-Werts wieder aus ihren Glucuroniden freigesetzt. Auch andere Enzymsysteme könnten zur Kanzerogenität von N-Acetylbenzidin beitragen. Es ist möglich, dass aus N′-Hydroxy-N-acetylbenzidin durch NAT1 der N′-Acetoxyester entsteht, der ebenfalls zur Krebsentstehung in der Harnblase beitragen kann (Carreón et al. 2006, 2007).

Basierend auf diesen Daten und den vorliegenden epidemiologischen Studien wird daher angenommen, dass eine langsame N-Acetylierung mit einem geringeren Risiko für Benzidin-induzierten Harnblasenkrebs einhergeht. Allerdings haben In-vitro-Experimente gezeigt, dass sowohl Benzidin als auch N-Acetylbenzidin bevorzugte Substrate für NAT1, nicht aber für NAT2 sind. Demnach sollte eine geringe NAT2-Aktivität (langsame Acetylierung) nicht die Benzidin-Acetylierung und die damit zusammenhängende Harnblasenkrebs-Inzidenz beeinflussen. Dies wird durch eine Studie an indischen Beschäftigten unterstützt, bei denen die urothelialen Addukte N-acetyliert, aber nicht mit NAT2-Aktivität, NAT2-Phänotyp oder NAT2-Genotyp assoziiert waren. Ein erhöhtes, nicht statistisch signifikantes Risiko für die Entstehung von Harnblasenkrebs wurde bei jenen Personen beobachtet, die homozygot oder heterozygot für NAT1*10 waren (Carreón et al. 2006; Pesch et al. 2013).

**Fazit:** Die Acetylierung durch NAT ist, im Gegensatz zum Metabolismus von Monoarylaminen, für Benzidin kein Entgiftungsschritt. Bei Benzidin erfolgt die Bildung von reaktiven Metaboliten, die für die Harnblasentumoren verantwortlich gemacht werden, vor allem durch diejenigen Benzidin-Metaboliten, die zunächst durch N-Acetylierung von Benzidin an der einen Aminogruppe via NAT und anschließender oxidativer Verstoffwechselung an der zweiten Aminogruppe entstehen. Die Daten legen nahe, dass sowohl Benzidin als auch N-Acetylbenzidin bevorzugte Substrate für NAT1, nicht aber für NAT2 sind. Demnach sollte eine geringe NAT2-Aktivität (langsame Acetylierung) nicht die Benzidin-Acetylierung und die damit zusammenhängende Harnblasenkrebs-Inzidenz beeinflussen.

**Abb. 1:**
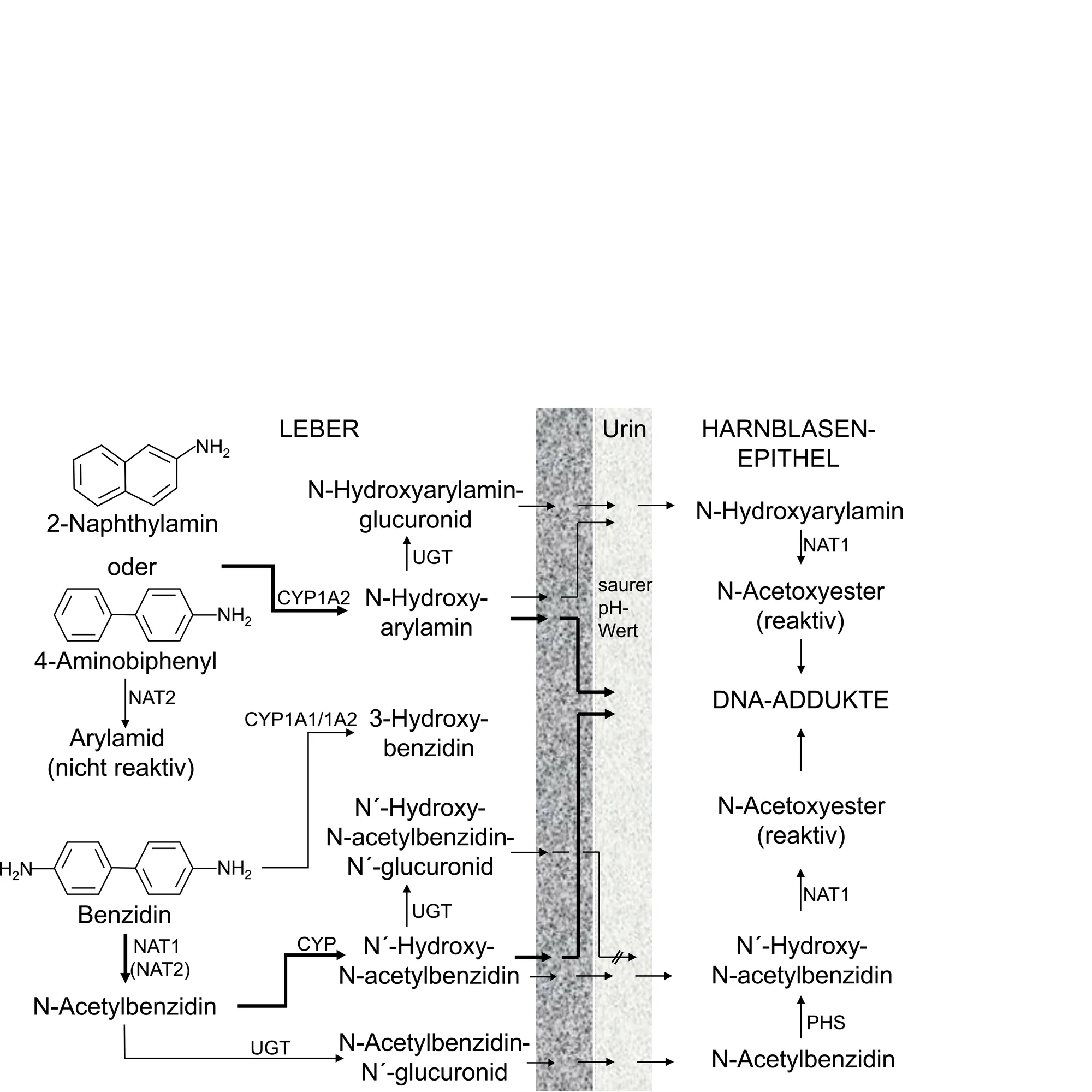
Metabolismus von Benzidin im Vergleich mit anderen kanzerogenen Arylaminen (nach Carreón et al. 2007)

CYP: Cytochrom-P450; NAT: N-Acetyltransferase; PHS: Prostaglandin-H-Synthase; UGT: UDP-Glucuronosyltransferase

## 3 Toxikokinetik und Metabolismus

Benzidin wird inhalativ und besonders auch dermal schnell resorbiert und in Form des freien oder konjugierten Amins und konjugierter Metaboliten mit Halbwertszeiten von bis zu sieben Stunden über die Nieren ausgeschieden (ATSDR 2001; Lewalter 2000). 

Die dermale Resorption wurde an männlichen F344-Ratten (225–250 g KG) untersucht. Sie erhielten auf 5–6 cm^2^ der rasierten Rückenhaut ^14^C-markiertes Benzidin (k. A. zur Reinheit; 1 mg/kg KG, gelöst in 0,2 ml Aceton). Nach 60 Minuten, 8 und 24 Stunden wurde die Radioaktivität in Organen, Restkörper, Urin und Faeces bestimmt. Nach den drei Zeitpunkten verblieben 94,6 %, 75,1 % bzw. 48,6 % an der Applikationsstelle. Nach einer Stunde wurden insgesamt in Leber, Darm und Restkörper ca. 4,4 % der applizierten Radioaktivität gefunden; nach acht Stunden ca. 4,1 % im Urin und ca. 0,7 % in den Faeces, nach 24 Stunden 22,8 % bzw. 18,7 % (Shah und Guthrie 1983). Aus der Resorption nach einer Stunde errechnet sich ein Flux von 1 mg/kg KG / 0,238 kg KG / 5,5 cm^2^ × 0,043 = 32,8 µg/cm^2^ und Stunde.

Die Verstoffwechselung der Diarylamine, wie Benzidin, verläuft anders als bei den Monoarylaminen. Benzidin ist im Gegensatz zu anderen aromatischen Aminen erst nach N-Acetylierung ein gutes Substrat für Cytochrom P450 (Leng et al. 2019). Benzidin wird also in der Leber zunächst zu N-Acetylbenzidin acetyliert und besitzt dann im Gegensatz zu Monoarylaminen eine weitere freie Aminogruppe, die für eine N′-Oxidation zu N′-Hydroxy-N-acetylbenzidin oder für eine N′-Glucuronidierung zu N-Acetylbenzidin-N′-glucuronid zugänglich ist. Auch kann die freie Aminogruppe durch die NAT weiter zu N,N′-Diacetylbenzidin acetyliert werden. Die Produkte der hepatischen Glucuronidierung oder Hydroxylierung werden in die Harnblase ausgeschieden, wo sie durch den sauren pH-Wert des Urins wieder gespalten werden können (Carreón et al. 2006, 2007; Abschnitt 2; Abbildung 1). 

## 4 Erfahrungen beim Menschen

In diesem Abschnitt sind nur Studien zu den bewertungsrelevanten Endpunkten Sensibilisierung, Genotoxizität und Kanzerogenität aufgeführt.

### Allergene Wirkung

Zur sensibilisierenden Wirkung von Benzidin liegen nur wenige Studien vor, da wegen der humankanzerogenen Wirkung praktisch keine Exposition mehr besteht. 

#### Allgemeine Studien

Von 100 Patienten, die positiv auf p-Phenylendiamin, Benzidin und Benzocain getestet wurden, reagierten in einer zweiten Testserie insgesamt elf auf nur eines der getesteten aromatischen Amine: vier davon auf Benzidin (k. A. zur Konzentration), vier bzw. drei Personen auf p-Phenylendiamin und Benzocain (k. w. A.). Bei den restlichen 89 Personen, die positiv auf mindestens zwei aromatische Amine reagierten, zeigten zwei ein identisches Sensibilisierungsmuster: Die Testergebnisse waren positiv auf Benzidin, p-Toluidin und p-Aminophenol, jedoch negativ auf weitere 29 aromatische Amine, auf Azobenzol und zwei Azofarbstoffe (Rudzki 1975). Der Grund für die Testung mit Benzidin wird nicht erläutert. Es liegen keine Informationen zu der eingesetzten Testkonzentration, den Ablesezeitpunkten, dem zeitlichen Verlauf und zur Reaktionsstärke vor. Daher wird diese Studie nicht zur Bewertung herangezogen.

Von 76 Patienten mit atopischer Dermatitis reagierten 1,3 % (n = 1) positiv auf 1 % Benzidin in Vaseline, bei 895 Patienten mit anderen Dermatitisformen (z. B. nummuläres Ekzem) betrug der Prozentsatz positiver Reaktionen 8,1 % (Rudzki und Grzywa 1975). Die Ablesung erfolgte 48 und 96 Stunden nach der Applikation (Rudzki und Kleniewska 1970). Auch in dieser Studie fehlen Angaben zum zeitlichen Verlauf und zur Stärke der Reaktionen.

#### Studien mit beruflicher Exposition

Von 4600 Patienten, die im Zeitraum von 1973 bis 1977 an eine dermatologische Klinik in Spanien verwiesen wurden, reagierten 927 (20,2 %) im Epikutantest positiv auf die getesteten „para-Verbindungen“ (u. a. Benzidin, p-Phenylendiamin, p-Aminophenol, Sulfonamide und die Lokalanästhetika Procain und Benzocain). Von den 927 wurden 231 positiv auf 3 % Benzidin in Vaseline (5 % des Gesamtkollektivs) getestet, wovon 153 (66 %) nur auf Benzidin, 78 (34 %) sowohl auf Benzidin als auch auf andere Allergene reagierten. Bei 38 (16,4 %) wurde eine Kreuzreaktion innerhalb der Gruppe der getesteten para-Verbindungen vermutet. Nach Angaben der Autoren trat die klinisch diagnostizierte Kontaktdermatitis bei 208 der 231 Patienten (66 Beschäftigte der metallurgischen Industrie und Maschinenbauindustrie, 55 Hausfrauen (Kontakt durch Waschen und Spülen gefärbter Baumwolltextilien), 41 Bauarbeiter, 20 Beschäftigte der Textilindustrie, 14 Friseure und 11 Beschäftigte der chemischen Industrie) auch im Zusammenhang mit ihrer beruflichen Tätigkeit auf. Allerdings scheint die einzige Gemeinsamkeit das Tragen von Kleidung oder Schuhen, die mit Benzidin-basierten Farbstoffen gefärbt worden sind, zu sein. Laut Autoren war die Verwendung dieser Farbstoffe in Spanien zum Zeitpunkt der Studie zwar zurückgegangen, aber noch nicht eingestellt (Grimalt und Romaguera 1981; Romaguera und Grimalt 1980). Es liegen keine Informationen zu den Ablesezeitpunkten, dem zeitlichen Verlauf und zur Reaktionsstärke vor. Für die positiven Reaktionen erscheint der Arbeitsplatzbezug fraglich. Ferner ist eine Kreuzreaktivität mit anderen aromatischen para-Aminoverbindungen wie p-Phenylendiamin möglich. 

In einer zwischen März und Dezember 2009 durchgeführten Querschnittstudie mit insgesamt 472 Beschäftigten zweier Gerbereien in Indonesien wurden anhand von Fragebogen-Interviews und Befundung der Haut 77 Personen mit aktueller Dermatitis identifiziert. Epikutantests wurden bei 63 dieser 77 Beschäftigten und bei 108 Gerbereibeschäftigten ohne Hauterkrankung als Kontrolle durchgeführt. Die Ablesezeitpunkte waren am 2., 4. und 7. Tag. Drei der 63 getesteten Personen (3,9 %) reagierten im Epikutantest mit 1 % Benzidin in Vaseline positiv, bei 13 mit aktueller arbeitsplatzbezogener Kontaktdermatitis wurden Reaktionen auf eines oder mehrere der insgesamt 15 getesteten Allergene festgestellt. Nur bei einem der drei Beschäftigten mit positiver Reaktion auf Benzidin wurde im Chemikalien-Verzeichnis der Gerberei ein Farbstoff auf Benzidin-Basis gefunden, was die Autoren als eine gegenwärtige relevante Exposition beschreiben. Bei den beiden anderen wird eine vorangegangene Exposition vermutet (Febriana et al. 2012 a, b, c). Es liegen keine Informationen zur Reaktionsstärke und zum Verlauf der Reaktion vor. Eine Testung gegen den in der Gerberei eingesetzten Farbstoff auf Benzidin-Basis ist offensichtlich nicht erfolgt. Es ist nicht nachvollziehbar, ob die positiven Reaktionen der drei Beschäftigten infolge der Exposition gegen einen Farbstoff auf Benzidin-Basis bzw. durch dessen Spaltung auf der Haut und der dadurch freigesetzten Bestandteile aus Benzidin zurückzuführen sind.

#### Fallberichte

Der im Folgenden dargestellte Einzelfallbericht beschreibt die Entwicklung einer allergischen Dermatitis in direktem Zusammenhang mit einer beruflichen Benzidin-Exposition. Ein 32-jähriger Mediziner mit starker, wiederkehrender ekzematöser Dermatitis an Händen und in schwächerer Ausprägung im Gesicht zeigte im Epikutantest mit Benzidin (als Feststoff) eine stark positive Reaktion. Die Dermatitis entwickelte sich immer nach der Durchführung der Benzidin-Probe zum Nachweis von Blut im Stuhl von Patienten. Epikutantests mit anderen Materialien, mit denen der Mediziner beruflich in Kontakt kam, verliefen negativ (Baer 1945).

### Genotoxizität

Die Untersuchung zytogenetischer Effekte bei 23 Arbeitern, die im Mittel 15 Jahre lang gegen Benzidin (0,42–0,86 mg/m^3^) und Benzidin-basierte Farbstoffe (7,8–32,3 mg/m^3^) exponiert worden waren, ergab eine statistisch signifikante Erhöhung an peripheren Lymphozyten mit Chromosomenaberrationen im Vergleich zu der Kontrollgruppe (ATSDR 2001).

### Kanzerogenität

Die krebserzeugende Wirkung von Benzidin an der Harnblase des Menschen ist nachgewiesen. Charakteristisch sind breitbasig aufsitzende oder gestielte Papillome, die karzinomatös entarten. Auch primäre Transitionalzellkarzinome werden beobachtet. Zahlreiche Fallberichte und Studien aus verschiedenen Ländern sind bezüglich der Harnblasentumoren beim Menschen nach Benzidin-Exposition publiziert und von verschiedenen Autoren und Gremien diskutiert und bewertet worden. Insgesamt zeigen die Fallberichte und epidemiologischen Untersuchungen einen starken und konsistenten Zusammenhang zwischen Benzidin-Exposition und dem erhöhten Harnblasenkrebsrisiko beim Menschen (ATSDR 2001, 2009; IARC 2012; Lewalter 2000; Nasterlack 2010; NTP 1980, 2021).

Folgende sind die wichtigsten Studienergebnisse in Stichpunkten aus IARC (2012):

- alle fünf einer Gruppe von Arbeitern, die mindestens 15 Jahre in der Benzidinherstellung beschäftigt waren, entwickelten Harnblasenkrebs
- bei 20 von 83 italienischen Arbeitern, die an der Herstellung und Verwendung von Benzidin in der Farbstoffindustrie beteiligt waren, wurden im Zeitraum von 1931 bis 1948 Harnblasentumore festgestellt
- zehn Todesfälle durch Harnblasenkrebs bei Arbeitern der Farbstoffindustrie, die nur Benzidin ausgesetzt waren (k. w. A.; standardisiertes Sterblichkeitsverhältnis (SMR) 13,9; 95-%-KI: 6,7–25,5)
- bei gegen Benzidin exponierten Arbeitern in der Farbstoffindustrie in China stieg die Sterblichkeit an Harnblasenkrebs mit zunehmender Dauer der Exposition (p für Trend < 0,01)
- in einer Kohorte von Arbeitern in der Benzidinherstellung in den USA war das Sterblichkeitsrisiko durch Harnblasenkrebs für diejenigen mit mindestens zwei Jahren Benzidin-Exposition deutlich erhöht (SMR 13,0; 95-%-KI: 4,8–28,4)
- in einer Kohorte von Arbeitern in der Farbstoffindustrie in Turin, Italien, betrug das SMR während der Exposition 100,8 und mindestens 20 Jahre nach Ende der Exposition 14,8
- bei Arbeitern einer chemischen Produktionsanlage in Shanghai, China, betrug das relative Risiko für Harnblasenkrebs nach Exposition gegen Benzidin für Nichtraucher 63,4 (p < 0,05) im Vergleich zu nicht exponierten Nichtrauchern; für Raucher ohne Exposition war das relative Risiko 6,2 (p = 0,05) und mit Exposition 152,3 (p < 0,01)
- bei chinesischen Arbeitern in der Herstellung und Verwendung von Benzidin lagen die Odds Ratios für Harnblasenkrebs bei 1,0; 2,7 (1,1–6,3) und 4,4 (1,8–10,8) für eine niedrige, mittlere bzw. hohe kumulative Benzidin-Exposition, nach Adjustierung für lebenslanges Zigarettenrauchen
- eine Fall-Kontroll-Studie aus Kanada zeigte ein übermäßiges Auftreten von Nierenzellkrebs in Abhängigkeit von der Dauer der Benzidin-Exposition (p < 0,004)

Folgende Studien sind nach der Bewertung der IARC (2012) publiziert worden: 

Ein 71-jähriger ehemaliger Arbeiter, der 29 Jahre in Baumwollfärbereien tätig war, davon drei Jahre als Färber, litt an einer Krebserkrankung der oberen Harnwege. Es wurde hauptsächlich Farbstoff auf Benzidin-Basis verwendet. Die Benzidin-Konzentration war nicht-nachweisbar bis 397,4 μg/m^3^. Der Krebs wurde 2018 mit einer Latenzzeit von etwa 35 Jahren diagnostiziert. Die Autoren sehen die Benzidin-Exposition für Krebserkrankungen nicht nur in der Harnblase, sondern auch in den oberen Harnwegen als wahrscheinlich ursächlich an (Kim et al. 2020).

In einer Kohorte von Arbeitern (n = 488), die in der letzten Benzidin-Produktionsanlage in den USA tätig waren, zeigte sich in einer bis zum Jahr 2014 durchgeführten Follow-up-Studie weiterhin ein erhöhtes Risiko an Harnblasenkrebs zu erkranken. Zum Zeitpunkt der Studie waren mindestens 41 Jahre seit der Exposition der Arbeiter vergangen. Sowohl Inzidenz als auch Mortalität waren statistisch signifikant erhöht (25 neu aufgetretene Fälle, standardisiertes Inzidenzverhältnis (SIR) 2,19 (95-%-KI: 1,42–3,23) und fünf Todesfälle, SMR 3,79 (95-%-KI: 1,23–8,84)). Es gab einen statistisch signifikanten Anstieg der Inzidenz und Mortalität bei denjenigen, die sowohl Benzidin als auch Dichlorbenzidin ausgesetzt waren (SIR 3,11 (95-%-KI: 1,97–4,67), SMR 4,10 (95-%-KI: 1,12–10,5)), aber nicht bei Arbeitern, die nur Dichlorbenzidin ausgesetzt waren (zwei neue Erkrankungsfälle, SIR 0,89 (95-%-KI: 0,11–3,23) und ein Todesfall, SMR 2,90 (95-%-KI: 0,07–16,15)). Die Inzidenz und Mortalität für Harnblasenkrebs war bei Arbeitern mit mehr als fünf Berufsjahren und nach mehr als 20 Jahren nach der letzten Exposition erhöht (sechs beobachtet, SIR 5,94 (95-%-KI: 2,18–12,92) und zwei Todesfälle, SMR 7,93 (95-%-KI: 0,96–28,65)) (Millerick-May et al. 2021).

Studien mit unklarer Exposition oder Ko-Exposition gegen andere Stoffe sind nicht aufgeführt.

## 5 Tierexperimentelle Befunde und In-vitro-Untersuchungen

In diesem Abschnitt sind nur Studien zu den bewertungsrelevanten Endpunkten Sensibilisierung, Genotoxizität und Kanzerogenität aufgeführt.

### Allergene Wirkung

Hierzu liegen keine Angaben vor.

### Genotoxizität

Es liegen zahlreiche In-vitro- und In-vivo-Studien vor, die die Genotoxizität von Benzidin belegen. Der Stoff verursachte Mutationen in Bakterien und genotoxische Effekte in verschiedenen Testsystemen in vitro in Human- und Säugerzellen. Bei Nagetieren induzierte Benzidin Mikronuklei, DNA-Strangbrüche, unplanmäßige DNA-Synthese, Chromosomenaberrationen, Schwesterchromatidaustausche und Aneuploidien (Details siehe ATSDR 2001, 2009). Bei trächtigen Mäusen zeigten sich nach intraperitonealer Gabe von Benzidin erhöhte Inzidenzen an Mikronuklei in der Leber der Feten, was vermuten lässt, dass Benzidin oder seine Metaboliten die Plazenta passieren (ATSDR 2001).

Es liegen weder neuere bewertungsrelevante Studien noch Studien an Keimzellen vor. In einer Studie mit lebenslanger Benzidindihydrochlorid-Gabe über das Trinkwasser traten bei Mäusen Effekte im Gehirn auf (spongiforme Leukenzephalopathie) (Littlefield et al. 1983; Morgan et al. 1981); damit ist die Überwindung der Blut-Hirn-Schranke gezeigt. Aufgrund dieser Daten kann für Benzidin und seine Salze die Passage der Blut-Testes-Schranke ebenfalls angenommen werden, und somit auch die Erreichbarkeit der Keimzellen.

### Kanzerogenität

Es gibt ausreichende Beweise für die Kanzerogenität von Benzidin am Tier. Die orale Verabreichung von Benzidin führte zu erhöhten Tumorinzidenzen an der Brustdrüse weiblicher Ratten, an der Leber von Mäusen und Hamstern und der Harnblase beim Hund. Nach subkutaner Verabreichung verursachte Benzidin Zymbaldrüsentumore bei Ratten und Lebertumore bei Mäusen, sowie nach intraperitonealer Verabreichung bei der Ratte Zymbal- und Brustdrüsentumore (IARC 1982, 1987; NTP 2021).

## 6 Bewertung

**Krebserzeugende Wirkung. **Die krebserzeugende Wirkung von Benzidin an der Harnblase des Menschen ist erwiesen. Charakteristisch sind breitbasig aufsitzende oder gestielte Papillome, die karzinomatös entarten. Auch primäre Transitionalzellkarzinome werden beobachtet. Zahlreiche Fallberichte und epidemiologische Studien aus verschiedenen Ländern zeigen einen starken und konsistenten Zusammenhang zwischen der Benzidin-Exposition und dem Harnblasenkrebsrisiko beim Menschen. Benzidin ist daher in die Kanzerogenitäts-Kategorie 1 eingestuft. Diese Einstufung wird beibehalten.

**Keimzellmutagene Wirkung. **Zahlreiche Studien belegen die genotoxische Wirkung von Benzidin in vitro und in vivo in Form von Mutationen, Klastogenität und Aneugenität. Es liegen keine Studien an Keimzellen vor. Die Überwindung der Blut-Hirn-Schranke bei Mäusen nach oraler Gabe von Benzidindihydrochlorid spricht auch für eine Passage der Blut-Testes-Schranke (Li et al. 2016; Wen et al. 2018). Somit ist davon auszugehen, dass Benzidin oder seine Metaboliten die Keimzellen erreichen. Daher werden Benzidin und seine Salze in die Kategorie 3 A für Keimzellmutagene eingestuft.

**Hautresorption. **Benzidin wird von Ratten nach dermaler Applikation in Aceton gut resorbiert. Der Stoff ist ein genotoxisches Kanzerogen und eine systemisch tolerable Dosis kann nicht angegeben werden. Deshalb bleibt die Markierung mit „H“ bestehen. Für Benzidinsalze liegen keine Untersuchungen vor. Vermutlich ist die Resorption geringer, kann aber nicht ausgeschlossen werden, sodass die Salze von Benzidin ebenfalls mit „H“ markiert bleiben.

**Sensibilisierende Wirkung. **Tierexperimentelle Untersuchungen oder Studien mit tierversuchsfreien Alternativverfahren fehlen. Die klinischen Befunde zeigen, dass vereinzelt positive Reaktionen auf Benzidin auftreten können. Eine eigenständige kontaktsensibilisierende Wirkung von Benzidin beim Menschen ist aus den vorliegenden klinischen Daten aufgrund methodischer Schwächen der zumeist älteren Studien nicht ableitbar. Da Patienten mit einer bestehenden Sensibilisierung gegen aromatische Aminoverbindungen, wie p-Phenylendiamin oder Anilin, häufig auch auf andere Verbindungen dieser Substanzklasse reagieren, ist eine Kreuzreaktivität auf Benzidin nicht auszuschließen. Aufgrund der vorliegenden Daten wird keine Markierung mit „Sh“ vorgenommen. Zur atemwegssensibilisierenden Wirkung von Benzidin und seinen Salzen liegen keine Daten vor, sodass keine Markierung mit „Sa“ erfolgt.

**MAK-Wert, Spitzenbegrenzung, Schwangerschaftsgruppe **Ein MAK-Wert wird nicht aufgestellt, daher entfällt auch die Spitzenbegrenzung und die Zuordnung zu einer Schwangerschaftsgruppe.
